# Investigation on Flavescence Dorée in North-Western Italy Identifies Map-M54 (16SrV-D/Map-FD2) as the Only Phytoplasma Genotype in *Vitis vinifera* L. and Reveals the Presence of New Putative Reservoir Plants

**DOI:** 10.3390/biology12091216

**Published:** 2023-09-07

**Authors:** Ivo Ercole Rigamonti, Martino Salvetti, Paola Girgenti, Piero Attilio Bianco, Fabio Quaglino

**Affiliations:** 1 Department of Food, Environmental and Nutritional Sciences, University of Milan, Via Celoria 2, 20133 Milan, Italy; ivo.rigamonti@unimi.it (I.E.R.); paola.girgenti@unimi.it (P.G.); 2 Fondazione Fojanini Di Studi Superiori, Via Valeriana 32, 23100 Sondrio, Italy; msalvetti@fondazionefojanini.it; 3 Department of Agricultural and Environmental Sciences—Production, Landscape, Agroenergy, University of Milan, Via Celoria 2, 20133 Milan, Italy; piero.bianco@unimi.it

**Keywords:** grapevine yellows, *Scaphoideus titanus*, *Orientus ishidae*, 16SrV, epidemiology

## Abstract

**Simple Summary:**

Flavescence dorée is one of the most important diseases of grapevines. It is associated with phytoplasmas, pathogenic bacteria transmitted from vine to vine by the insect vector *Scaphoideus titanus*. The disease spread is limited by eradication of affected vines and control of *S. titanus* populations using insecticides. Recent studies highlighted that epidemic (transmitted by *S. titanus*) and non-epidemic phytoplasma strains can infect grapevines, and other insects and additional host plants are involved in Flavescence dorée epidemiology, increasing the risk of disease outbreaks. Thus, the objective of the present study was to identify the phytoplasma strains infecting grapevines in north-western Italy and investigate the presence of additional hosts. Only one epidemic phytoplasma strain was found in diseased grapevines. Moreover, this and other phytoplasma strains, reported as epidemic strains in previous studies, were found in several additional host plants and insect vectors. These findings reinforced the idea that Flavescence dorée epidemiological patterns are not limited to within vineyards, but they involve the entire ecosystems in which the vineyards are located.

**Abstract:**

Flavescence dorée (FD) is the most important phytoplasma-associated disease of the grapevine yellows complex in Europe. Recent studies highlighted a great genetic diversity within FD phytoplasma (FDp) strains and demonstrated that their diffusion is not related exclusively to the pathosystem including *Vitis vinifera* L. and *Scaphoideus titanus* but involves additional vectors and reservoir plants. This study aimed to investigate FD epidemiology in north-western Italy, with a particular focus on FDp hosts. During field surveys, leaf samples were collected from symptomatic grapevines and other symptomless plant species, and insects were collected within and around vineyards. Phytoplasmas belonging to the ribosomal group 16SrV were detected and typed using nested-PCR-based amplification and nucleotide sequence analyses of the *map* gene. All symptomatic grapevines were found to be infected by the FDp genotype M54, prevalent in *S. titanus* and also identified in other known and newly reported hosts. Interestingly, other FDp strains (M38, M50, M51, M121) and FDp-related strains (M39, M43, M48), never detected in grapevines, were largely identified in several known and newly reported host plants and insects including *S. titanus*. Such evidence confirmed the complexity of FD ecology, expanding the knowledge on the range of FDp host plants putatively involved in the disease spread.

## 1. Introduction

In Europe, Flavescence dorée (FD) is the only epidemic and the most economically damaging disease of the grapevine yellows complex (GY) [1]. FD causes symptoms in different organs of the vine (leaf, branch, bunch); these symptoms include (i) chromatic alteration of the leaf lamina (including the veins) and rolling/curling of the leaf margins, (ii) lack of lignification and appearance of blackish pustules on the shoots, and (iii) dehydration, desiccation (June/July), and withering (August/September) of the bunches [2]. FD is associated with a phytoplasma (Flavescence dorée phytoplasma (FDp); taxonomic subgroups 16SrV-C and -D) transmitted from vine to vine by the vector *Scaphoideus titanus* Ball, which completes its life cycle only on vines [3,4]. Since FD is a highly epidemic disease, FDp is a quarantine pathogen, the containment of which is regulated by a mandatory control decree including insecticide treatments on the *S. titanus* populations and the uprooting of symptomatic vines [2]. In recent years, several studies (i) highlighted an extensive genetic diversity among FDp and FDp-related strain populations based on nucleotide sequence analyses of variable genes (e.g., *map* and *vmp*) [5,6,7,8,9,10], (ii) evidenced that epidemic FDp strains are transmitted from vine to vine by *S. titanus*, and some of them can be moved from additional plant hosts (*Alnus glutinosa*, *Clematis vitalba*) to grapevines by alternative vectors (*Dictyophara europaea*, *Orientus ishidae*, *Allygus mixtus*) [9,11], (iii) demonstrated that FDp-related strains can be moved from alder to grapevines by the vector *Oncopsis alni*, but are not transmitted by *S. titanus* (non-epidemic strains associated with Palatinate grapevine yellows, PGYs) [9], (iv) reported putative vectors (*Phlogotettix cyclops*, *Hishimonus hamatus*) able to acquire FDp in controlled conditions [9,12,13], and (v) highlighted a continuously increasing list of FDp host plants (e.g., *Ailanthus altissima*, *Corylus avellana*, *Salix* spp.) [6,10]. This evidence demonstrated that the FD epidemiology is not restricted to the close pathosystem “grapevine-*S. titanus*”, but also involves additional vectors and reservoir plants. Alternative vectors, which are not capable of transmitting FDp from vine to vine, in any case guarantee the continuous presence of FDp within the vineyard, even thought this may be sporadic, allowing its possible rapid spread on grapevines by *S. titanus*. Interestingly, this fact, along with the withdrawal of broad-spectrum insecticides used in viticulture against leafhoppers, may contribute to explaining the frequent phenomena of FD recrudescence reported in recent years. These data reinforced the evidence of a dynamic evolution of the FD epidemiology scenario, underlying its ever-increasing complexity [6,9,10]. Therefore, the study of host plants, potential vectors, and FDp strains present in the vineyard agro-ecosystem constitutes the starting point for obtaining information on FD epidemiology and planning adequate intervention strategies.

The aim of the present study was to investigate the FD epidemiological cycle in north-western Italy by analyzing the diffusion and the genetic diversity of FDp and FDp-related strains in host plants (grapevine and wild plant species) and in known and putative insect vectors.

## 2. Materials and Methods

The experimental activities were carried out in two years (2019 and 2021) with different objectives. Considering the results obtained by Casati et al. [6], in 2019, the study was focused on the presence of FDp additional host plants in two vineyards localized in the Lombardy and Piedmont regions; in 2021, the activity, conducted at the landscape level in the Valtellina grapevine growing area (Lombardy region), aimed to look for (i) new FDp host plants and (ii) new insect vectors. Genetic characterization of all detected 16SrV phytoplasma strains was carried out through nucleotide sequence analyses of the *map* gene as reported by Malembic-Maher et al. [9].

### 2.1. Surveys and Sample Collection in Vineyards and Surroundings

In September 2019, the sampling activities were carried out in two vineyards localized in Mombaruzzo (Asti province, Piedmont region) (44°45′40″ N/8°24′20″ E) and Gussago (Brescia province, Lombardy region) (45°25′25″ N/10°10′15″ E) to survey the presence of grapevine yellows and additional host plants of FDp and FDp-related strains. These sites were selected for the presence of susceptible varieties (Barbera in Mombaruzzo, Chardonnay in Gussago) and the recent reports of FD recrudescence in northern Italy in recent years [8,14]. Leaf samples and petioles were collected from 31 and 22 grapevines showing typical GY symptoms in Mombaruzzo and Gussago vineyards, respectively. Moreover, leaf samples were collected from the wild woody and shrubby plant species more prevalent in the vineyard surroundings. In detail, samples from 76 plants of 12 species and from 58 plants of 12 species were collected in Mombaruzzo and Gussago, respectively (Table 1).

In 2021, the activities were conducted in the Valtellina vine growing area (Lombardy, northern Italy) with the aim of monitoring the presence of grapevine yellows, *S. titanus*, and other known and putative FDp vectors in the vineyards and on wild plants. Valtellina is an alpine valley situated in the northernmost part of Lombardy, near the Swiss border. The climate is sub-continental temperate with an annual mean temperature of 12.2 °C and total annual rainfall of 947 mm (Sondrio weather station, Fojanini Foundation). The grapevine cultivated area extends over 50 km on the slopes of the Retiche Alps for a total of 820 ha and is isolated from the nearest viticultural areas by the Orobie Alps. The vineyards are planted almost exclusively with Nebbiolo, locally known as Chiavennasca, a variety less susceptible to FD. Woody lots are interspersed among the vineyards and woods are common on the mountain slopes and along the Adda river. FD was first detected in Valtellina only in 2002 and its incidence was always low [2]. These characteristics make Valtellina an interesting site to survey the diffusion and genetic diversity of FDp and FDp-related strains at the landscape level.

We sampled 37 vineyards (V1–V37, Table S1) and 15 woody areas (W1–W15, Table S2). The vineyards were chosen to cover all the main viticultural areas, while the woody sites were chosen on the basis of (i) the presence of known FDp plant hosts and (ii) the presence of the most common plant species of the area. In September, leaf samples and petioles were collected from all GY-symptomatic grapevines (mainly Nebbiolo variety) found in the monitored area. Some of these samples were collected based on the annual official survey data provided by the Regional Phytosanitary Service. On 20 July and 22 September, leaf samples were collected from 139 wild plants belonging to 10 species in the vineyard surroundings (Table 1).

At each of the 52 sites (vineyards and woody areas), one 25 cm × 10 cm, Glutor^®^ yellow sticky trap (CBC Europe S.r.l.-BIOGARD DIVISION, 24050 Grassobbio (BG), Italy) was placed vertically in the canopy. The monitoring activities started on 9 July, and the traps were collected and substituted biweekly until the end of September then were stored at −20 °C until examination. Auchenorrhyncha specimens, after being sorted out from the material caught in the traps, were individually identified at the species level with a stereomicroscope. All individuals of species belonging to the subfamily Deltocephalinae, considered putative vectors of FDp [15], and other leafhopper and planthopper species, reported in the scientific literature as known vectors of phytoplasmas, were preserved in 70% ethanol for further molecular analyses.

### 2.2. 16SrV Phytoplasma Detection

Total nucleic acids (TNAs) were extracted from 1 g of leaf petioles and/or veins of the collected plant samples and from collected insect specimens using extraction protocols previously published by Angelini et al. and Marzachì et al. [16,17], respectively. Extracted TNAs were resuspended in TE buffer (150 μL for each plant sample; 40 μL for each insect specimen), checked for their quality and quantity using a Nanodrop system, and stored at −20 °C until molecular analyses. Nested PCR reactions were carried out on extracted TNAs (50 to 100 ng) to specifically detect the presence of 16SrV phytoplasmas by amplifying the *map* gene. PCR reactions were conducted employing the primer pair FD9F5/MapR1 (direct PCR), followed by the primer pair FD9F6/MapR2 (nested PCR), as previously described by Arnaud et al. [5]. In PCRs, TNAs extracted from *Catharanthus roseus* L. (G. Don) (periwinkle) plants, maintained in an insect-free greenhouse at University of Milan and infected by phytoplasma strains EY1 (‘*Ca*. P. ulmi’, 16SrV-A; 16S rDNA sequence Acc. No. AY197655) and STOL (‘*Ca*. P. solani’, 16SrXII-A; 16S rDNA sequence Acc. No. AF248959), were used as reference controls. Reaction mixtures devoid of DNA were used as negative controls. PCR reaction results were verified through 1% agarose gel electrophoresis in TBE buffer and visualized under a UV transilluminator.

### 2.3. Molecular Typing and Evolutionary Relatedness of 16SrV Phytoplasma Strains

FD9F6/MapR2 amplicons were sequenced in both strands (2× coverage per base position) by a commercial sequencing service (Eurofins Genomics, Ebersberg, Germany). Obtained nucleotide sequences were assembled by the Contig Assembling Program and trimmed to the annealing sites of the FD9F6 and MapR2 primers in the software BioEdit version 7.2.6 [18]. *Map* gene nucleotide sequences, obtained in this study from 16SrV phytoplasma strains identified in grapevines, alternative plant hosts, and insects, were aligned using the ClustalW Multiple Alignment program in the software BioEdit and analyzed using Sequence Identity Matrix to calculate their genetic diversity. Finally, *map* sequence variants were aligned with representative sequences of previously defined *map* genotypes (Table S3) [5,6,7,8,9,10,11,19,20].

Evolutionary relatedness and genealogy of the *map* genotypes, identified in this and in previous studies (Table S3) [5,6,7,8,9,10,11,19,20], were defined through genotype networks generated using the software PopART version 1.7 (http://popart.otago.ac.nz; accessed on 7 June 2023) by performing median-joining (MJ) calculation, maintaining the parameter e = 0. Two networks were generated: (i) an overall network covering the *map* genotypes described in this and previous studies, including their biological features, and (ii) a network comprising exclusively the *map* genotypes identified in this study, indicating their frequencies in the associated hosts.

## 3. Results

### 3.1. Phytoplasmas Identified in Grapevines and Wild Plants

PCR amplification of the *map* gene identified the presence of 16SrV phytoplasmas in 50 out of 88 overall symptomatic grapevines. The percentage of 16SrV-phytoplasma-infected grapevines was from 46% (Gussago and Valtellina) to 77% (Mombaruzzo) (Table 1). Due to the presence of 38 symptomatic grapevines negative for 16SrV phytoplasmas, further analyses were carried out to detect the presence of ‘*Candidatus* Phytoplasma solani’ (CaPsol), associated with bois noir (BN) [21], through the PCR-based amplification of the *stamp* gene as previously described [22]. In total, 10 out of 88 symptomatic grapevines (11% in Valtellina, 14% in Gussago, and 9% in Mombaruzzo) were found to be infected by CaPsol, indicating the lower incidence of BN in the examined areas. No mixed infections were found.

Considering wild plants, 16SrV phytoplasmas were detected in 12 out of the 20 examined species; in detail, 71 out of the 273 plants collected in 2019 and 2021 in the three considered areas were found to be infected, with an overall infection rate of 26%. The infection rate in the three examined areas was 33% in Valtellina (46 out of 139 plants; 6 species out of 10), 22% in Gussago (13 out of 58 plants; 6 species out of 12), and 15% in Mombaruzzo (12 out of 76 plants; 6 species out of 12) (Table 1). Three wild species were present in all the sampled areas; *Clematis vitalba* (28 plants) was found to always be negative through PCR amplification, while *Juglans regia* (4 out of 30 plants) and *Sambucus nigra* (5 out of 13) were found to be infected in two out of the three areas. Eight wild species were present in two of the sampled areas: gone-wild *Vitis vinifera* (8 plants) and *Prunus avium* (3 plants) was found to always be negative; *Cornus sanguinea* (1 out of 4) and *Rubus ulmifolius* (1 out of 29) were found to be infected in one area; and *Ailanthus altissima* (13 out of 20), *Carpinus betulus* (2 out of 5), *Corylus avellana* (10 out of 51), and *Robinia pseudoacacia* (5 out of 36) were found to be positive in both areas. The remaining nine species were observed and collected only in one area: *Celtis australis* (2 plants), *Mespilus germanica* (1 plant), *Populus* spp. (4 plants), *Prunus armeniaca* (1 plant), and *Quercus* sp. (2 plants) were negative; *Alnus glutinosa* (21 out of 23), *Prunus cerasifera* (1 out of 3), *Prunus domestica* (6 out of 8), and *Salix* spp. (2 out of 2) were found to be infected (Table 1).

Bioinformatics analyses revealed that 16SrV phytoplasma strains, identified in 50 infected grapevines, shared an identical *map* gene nucleotide sequence, undistinguishable from the sequence of the FDp strain M54 (Acc. No. AM384886) (Table 2). Analyses of the *map* gene sequences obtained from the 66 16SrV phytoplasma strains, identified in infected wild plants, revealed the presence of 10 sequence variants identical to those previously reported for FDp strains [M38 (Acc. No. LT221933), M50 (Acc. No. AM384887), M51 (Acc. No. FN811141), M54 (Acc. No. AM384886), M121 (Acc. No. LT222016)], FDp-related strains [M39 (Acc. No. LT221934), M43 (Acc. No. AM384890), M48 (Acc. No. AM384893)], and strains not related to FD [M58 (Acc. No. LT221953), M78 (Acc. No. LT221973)]. Moreover, five phytoplasma strains share a *map* gene sequence variant identical to the ‘*Ca*. Phytoplasma ulmi’ strain E04-D714 (GenBank Acc. No. AM384901). The FDp strain M54, identified in all infected grapevines, was found in seven plants of five wild species: *A. altissima* (one plant), *C. avellana* (three plants), *R. pseudoacacia* (one plant), and *S. nigra* (one plant) in Mombaruzzo, and in *J. regia* (one plant) in Gussago. Among the FDp strains detected exclusively in wild plants, the most abundant was M50, identified in 29 plants of six species from Mombaruzzo and Valtellina, followed by M51 (identified in 9 plants of five species from Gussago), M38 (3 plants of three species from Valtellina), and M121 (1 plant from Valtellina) (Table 2). Among the FDp-related (non-epidemic) strains detected exclusively in wild plants, the most abundant was M39, identified in six plants of four species from Valtellina, followed by M43 (identified in four plants of two species from Valtellina) and M48 (identified in two plants of *Alnus glutinosa* from Valtellina) (Table 2). Strains not related to FD were identified exclusively in three plants of two wild species from Valtellina (M78) and in two plants of *A. glutinosa* from Valtellina (M58) (Table 2). Strains characterized by the ‘*Ca*. P. ulmi’ *map* variant were identified in five plants of three wild species from Gussago and Mombaruzzo (Table 2).

**Figure 1:**
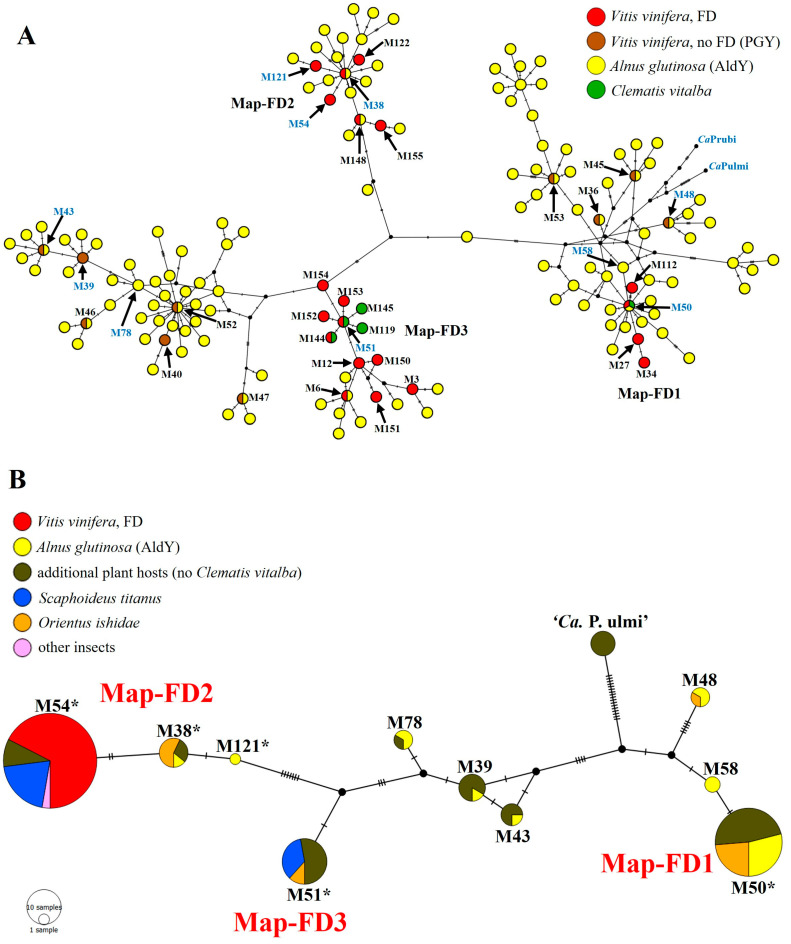
Median-joining networks inferred from *map* genotypes of FDp and related strains. Geno-types are represented by circles and paired genotypes are connected by a line. Each SNP mutation is represented by a hatch mark, while black dot vertices represent median vectors. (**A**) Network including 157 *map* genotypes present in the literature (genotypes identified in the present study are written in blue). (**B**) Network including *map* genotypes identified in the present study in plants and insects (circle diameter is related to the number of samples infected by distinct *map* genotypes; asterisks indicate genotypes reported in the literature as epidemic FDp strains).

Network analyses based on *map* gene sequence analysis highlighted that among the 16SrV phytoplasmas identified in plants in this study, (i) FDp strains M54, M38, and M121 belong to the cluster Map-FD2, including other two FDp strains (M148 and M155) recently reported in Serbia [10], (ii) FDp strain M50 belongs to the cluster Map-FD1, including three other FDp strains (M27, M34, M112) identified in France [9], (iii) FDp strain M51 belongs to the cluster Map-FD3, including other 11 FDp strains (M3, M6, M12, M119, M144, M145, M150, M151, M152, M153, M154) previously reported in Italy, Montenegro, Serbia, and Switzerland [6,8,19,20], (iv) FDp-related (M39, M43) and -unrelated (M78) strains belong to a cluster closely related to Map-FD3, and (v) FDp-related (M48), FDp-unrelated (M58), and ‘*Ca*. P. ulmi’ strains belong to a cluster closely related to Map-FD1 (Figure 1).

### 3.2. Phytoplasmas Identified in Insects

In the 37 surveyed vineyards, insect specimens belonging to four species were captured. The most abundant were *Scaphoideus titanus* (45 specimens from 16 vineyards) and *Orientus ishidae* (25 specimens from 13 vineyards); moreover, 1 specimen each of *Fieberiella florii* and *Neoaliturus fenestratus* was captured. In the 15 woody areas, insect specimens belonging to four species were captured. The most abundant were *O. ishidae* (60 specimens from 10 areas) and *S. titanus* (9 specimens from 3 areas); moreover, 2 specimens of *F. florii* from 2 areas and 1 specimen of *Macrosteles* sp. were captured.

PCR-based amplification of the *map* gene allowed the detection of 16SrV phytoplasmas in 39 out of 144 insect specimens, with an overall infection rate of 27%. In detail, 21 out of the 72 specimens captured within vineyards (infection rate 28%) and 18 out of the 72 specimens captured outside vineyards (infection rate 25%) were found to be infected. The highest infection rate (39%) was found in *S. titanus* (21 specimens infected out of 54), followed by *O. ishidae* (16 out of 85 specimens; infection rate 19%). Moreover, both *N. fenestratus* and *Macrosteles* sp. specimens were found to be infected, while the three *F. florii* specimens were negative in PCR reactions. The infection rates of *S. titanus* and *O. ishidae* within and outside vineyards were different: (i) for *S. titanus*, the infection rate was 44% within and 11% outside vineyards; (ii) for *O. ishidae*, it was zero within and 26% outside vineyards (Table 1).

Nucleotide sequence analyses of *map* gene amplicons obtained from 39 16SrV phytoplasma strains, identified in infected insects, revealed the presence of five sequence variants identical to those previously reported for FDp strains (M38, M50, M51, M54) and the FDp-related strain M48 (Table 2). The FDp strain M54, identified in all infected grapevines and found as the most frequent strain in insects, was present in 17 specimens of three species: *S. titanus* (14 specimens within and 1 outside vineyards), *N. fenestratus* (1 specimen within a vineyard), and *Macrosteles* sp. (1 specimen outside a vineyard). Among the other FDp strains, M50 was found in nine *O. ishidae* specimens, M51 in eight specimens of two species (six specimens of *S. titanus* within vineyards, and two of *O. ishidae*), and M38 in four *O. ishidae* specimens. The FDp-related strain M48 was identified in one *O. ishidae* specimen (Table 2). The FDp epidemic strain M121, the FDp-related strains M39 and M43, and the strains not related to FD (M58, M78) were not found in the analyzed insects. Evolutionary relatedness, *map* cluster attribution, and host prevalence of *map* genotypes identified in insects are as described above for phytoplasmas identified in plants and shown in Figure 1.

## 4. Discussion

The obtained results highlighted the higher prevalence of FD compared to BN in the examined areas. However, around 30% of symptomatic grapevines were negative for both 16SrV and 16SrXII-A phytoplasmas. This result can be reasonably explained by different hypotheses: (i) the uneven distribution of phytoplasmas in the phloem tissues of infected plants (Constable et al., 2003) [23], (ii) the possible presence of other phytoplasmas associated with grapevine yellows in Italy (Zambon et al., 2019) [24], and (iii) concerning the Nebbiolo variety (Valtellina), the presence of mild symptoms observed on the canopy is often associated with a low titer of the pathogen in the phloem tissue (Roggia et al., 2014) [25].

The molecular characterization of 16SrV phytoplasma strains identified in symptomatic grapevines from Piedmont and Lombardy highlighted the exclusive presence of the FDp genotype M54 (16SrV-D/Map-FD2), confirming its large prevalence in north-western Italy and Canton Ticino (Switzerland) [6,8,20]. It is remarkable that all gone-wild grapevines sampled in the investigated area were found to not be infected by FDp. Even though the number of analyzed samples was limited, this seems to indicate a different epidemiological scenario compared with the situation previously reported in Piedmont, where gone-wild grapevines were found to be a main FDp inoculum source for *Scaphoideus titanus* [8,26]. Based on the evidence from previous studies, the epidemiological pattern of the FDp genotype M54 is strictly limited to the close system ‘*S. titanus*-grapevine’, even if it was occasionally reported in additional host plants (*Corylus avellana* and herbaceous plants) and insects (*Orientus ishidae* and *Thamnotettix dilutior*) [6,27]. Data from the present study confirmed the occasional presence of FDp M54 in *C. avellana* and reported it for the first time in *Ailanthus altissima*, *Juglans regia*, *Robinia pseudoacacia*, and *Sambucus nigra*. Moreover, genotype M54 was also identified in *Neoaliturus fenestratus* and *Macrosteles* sp. in Valtellina. All this information proved the increasing presence of FDp M54 additional hosts (plants and insects) in the vineyard agro-ecosystem. This new evidence led to the necessity of in-depth investigation of the possible role of such hosts in an open epidemiological cycle.

Interestingly, concerning other FDp strains, data from this study showed quite a different ecological cycle compared to that previously reported. Regarding the FDp genotype M51 (16SrV-C/Map-FD3), related to the epidemiological cycle including *Clematis vitalba*/*Ailanthus altissima* (source) and *Dictyophara europaea* (vector) [9,10,11], it was never detected in these hosts, but it was identified in five newly reported plant hosts (*Cornus sanguinea*, *J. regia*, *Prunus cerasifera*, *P. domestica*, *S. nigra*) in the Brescia province only (Table 2). Moreover, in Valtellina, M51 was detected only on *S. titanus* specimens captured within vineyards and *O. ishidae* collected outside vineyards. Considering that (i) all symptomatic grapevines from Valtellina were analyzed and the genotype M51 was never detected and (ii) *S. titanus* feeds in controlled conditions on different plants [4,28,29], it can be hypothesized that *S. titanus*, captured within Valtellina vineyards, acquired this FDp strain from an unknown source. Moreover, the report of some known *O. ishidae* host plants (*C. sanguinea*, *J. regia*, *P. domestica*) [30] among the newly identified hosts of the genotype M51 suggests their role as putative reservoir plants.

Concerning the FDp genotypes M38 (16SrV-C/Map-FD2) and M50 (16SrV-C/Map-FD1), related to the epidemiological cycle including *Alnus glutinosa* (source) and *Allygus mixtus* and *Orientus ishidae* (vectors) [9], the obtained results confirmed the literature data and allowed their identification on five additional plant hosts (*A. altissima*, *C. avellana*, *J. regia*, *R. pseudoacacia*, *S. nigra*) in Piedmont and Valtellina (Table 2). Due to the polyphagyia of *O. ishidae* and its high infection rate outside vineyards (Table 2) [30], it is reasonable to hypothesize its role in the spread of such FDp genotypes at the landscape level. Moreover, the genotype M121 (16SrV-C/Map-FD2), previously identified exclusively in FD-affected grapevines [9], was found only in alder in Valtellina, suggesting its possible open epidemiology.

Finally, the PGY-associated phytoplasma (FDp-related) strains M39, M43, and M48, identified in *A. glutinosa* and additional hosts (*A. altissima*, *C. avellana*, *Salix* spp.) in Valtellina, were reported in Italy for the first time, where only the PGY-associated strains M36 and M53 were reported in previous studies [9].

Interestingly, ‘*Ca*. Phytoplasma ulmi’-related strains, known to infect different species of the *Ulmus* genus (https://gd.eppo.int/taxon/PHYPUL/hosts; accessed on 15 June 2023), were found in three additional host plants (*Carpinus betulus*, *P. domestica*, *R. ulmifolius*) in north-western Italy (Table 2).

## 5. Conclusions

The findings of the present study generally confirmed the dynamic situation of the FD epidemiological scenario, further enlarging the range of host plants putatively involved in FDp spread, leading to an increasing risk of disease outbreak.

## Figures and Tables

**Table 1:** 16SrV phytoplasma-infected grapevines, additional plant hosts, and insects (in: inside vineyard; out: outside vineyard).

Host	No. of 16SrV-Phytoplasma-Infected/Collected Samples		
	Gussago (BS)	Mombaruzzo (AT)	Valtellina (SO)
	2019	2019	2021
*Vitis vinifera*	10/22	24/31	16/35
*Vitis vinifera* gone wild		0/4	0/4
*Ailanthus altissima*		1/4	12/16
*Alnus glutinosa*			21/23
*Carpinus betulus*	1/3	1/2	
*Celtis australis*	0/2		
*Clematis vitalba*	0/7	0/6	0/15
*Cornus sanguinea*	1/3	0/1	
*Corylus avellana*		4/30	6/21
*Juglans regia*	3/9	0/5	1/16
*Mespilus germanica*	0/1		
*Populus* spp.			0/4
*Prunus armeniaca*	0/1		
*Prunus avium*	0/2	0/1	
*Prunus cerasifera*	1/3		
*Prunus domestica*	6/8		
*Quercus* sp.		0/2	
*Robinia pseudoacacia*		1/6	4/30
*Rubus ulmifolius*	0/18	1/11	
*Salix* spp.			2/2
*Sambucus nigra*	1/1	4/4	0/8
*Fieberiella florii* in			0/1
*Fieberiella florii* out			0/2
*Macrosteles* sp. out			1/1
*Neoaliturus fenestratus* in			1/1
*Orientus ishidae* in			0/25
*Orientus ishidae* out			16/60
*Scaphoideus titanus* in			20/45
*Scaphoideus titanus* out			1/9

**Table 2:** *map* (M) genotypes of 16SrV phytoplasmas identified in plants and insects (in: inside vineyard; out: outside vineyard). Origin: BS (Gussago), AT (Mombaruzzo), SO (Valtellina).

Host	Origin	16SrV Phytoplasma Strain										
	Year	M38	M39	M43	M48	M50	M51	M54	M58	M78	M121	*Ca.* P. ulmi
*Vitis vinifera*	BS, 2019							10				
	AT, 2019							24				
	SO, 2021							16				
*Ailanthus altissima*	AT, 2019							1				
	SO, 2021		1	3		7				1		
*Alnus glutinosa*	SO, 2021	1	1	1	2	11			2	2	1	
*Carpinus betulus*	BS, 2019											1
	AT, 2019											1
*Cornus sanguinea*	BS, 2019						1					
*Corylus avellana*	AT, 2019					1		3				
	SO, 2021	1	2			3						
*Juglans regia*	BS, 2019						2	1				
	SO, 2021					1						
*Prunus cerasifera*	BS, 2019						1					
*Prunus domestica*	BS, 2019						4					2
*Robinia pseudoacacia*	AT, 2019							1				
	SO, 2021	1				3						
*Rubus ulmifolius*	AT, 2019											1
*Salix* spp.	SO, 2021		2									
*Sambucus nigra*	BS, 2019						1					
	AT, 2019					3		1				
*Macrosteles* sp. out	SO, 2021							1				
*Neoaliturus fenestratus* in	SO, 2021							1				
*Orientus ishidae* out	SO, 2021	4			1	9	2					
*Scaphoideus titanus* in	SO, 2021						6	14				
*Scaphoideus titanus* out	SO, 2021							1				

## Data Availability

The data presented in this study are available in the article and its Supplementary Materials.

## Supplementary Materials

The following supporting information can be downloaded at: https://www.mdpi.com/article/10.3390/biology12091216/s1, Table S1: Position (coordinates) of monitored vineyards in Valtellina; Table S2: Position (coordinates) of monitored woody areas in Valtellina; Table S3: 16SrV phytoplasma strains (references [7,8,9,10,11,19,20]) used in median-joining network analyses.
