# Supplementary material for: Investigation on Flavescence Dorée in North-Western Italy Identifies Map-M54 (16SrV-D/Map-FD2) as the Only Phytoplasma Genotype in Vitis vinifera L. and Reveals the Presence of New Putative Reservoir Plants
Source: Biology (Basel). 2023 Sep 7;12(9):1216. doi: 10.3390/biology12091216 (PMC10525977; doi:10.3390/biology12091216)
Supplement: Supplementary file 1 [file biology-12-01216-s001.zip › biology-2524749-supplementary.pdf]

Table S1. Position (coordinates) of monitored vineyards in Valtellina.

Site	Latitude N	Longitude E	Site	Latitude N	Longitude E	Site	Latitude N	Longitude E
V1	46° 10’ 38”	9° 41’ 41”	V14	46° 10’ 26”	9° 54’ 05”	V27	46° 10’ 11”	10° 00’ 36”
V2	46° 10’ 37”	9° 41’ 45”	V15	46° 10’ 26”	9° 54’ 12”	V28	46° 10’ 08”	10° 00’ 45”
V3	46° 10’ 04”	9° 47’ 22”	V16	46° 10’ 30”	9° 54’ 25”	V29	46° 10’ 10”	10° 00’ 48”
V4	46° 10’ 02”	9° 49’ 05”	V17	46° 10’ 23”	9° 55’ 21”	V30	46° 10’ 07”	10° 00’ 57”
V5	46° 10’ 04”	9° 49’ 05”	V18	46° 10’ 27”	9° 55’ 32”	V31	46° 09’ 39”	10° 03’ 42”
V6	46° 10’ 00”	9° 49’ 52”	V19	46° 10’ 29”	9° 55’ 46”	V32	46° 10’ 06”	10° 03’ 56”
V7	46° 10’ 02”	9° 50’ 26”	V20	46° 10’ 35”	9° 55’ 57”	V33	46° 09’ 41”	10° 02’ 58”
V8	46° 09’ 51”	9° 50’ 34”	V21	46° 10’ 39”	9° 56’ 54”	V34	46° 09’ 41”	10° 03’ 05”
V9	46° 10’ 39”	9° 52’ 54”	V22	46° 10’ 39”	9° 56’ 55”	V35	46° 09’ 48”	10° 04’ 24”
V10	46° 10’ 23”	9° 53’ 23”	V23	46° 10’ 39”	9° 56’ 57”	V36	46° 11’ 12”	10° 06’ 16”
V11	46° 09’ 15”	9° 50’ 04”	V24	46° 10’ 10”	10° 00’ 32”	V37	46° 12’ 12”	10° 07’ 42”
V12	46° 10’ 45”	9° 53’ 26”	V25	46° 10’ 09”	10° 00’ 31”			
V13	46° 10’ 25”	9° 53’ 44”	V26	46° 10’ 07”	10° 00’ 39”			

Table S2. Position (coordinates) of monitored woody areas in Valtellina.

Site	Latitude N	Longitude E	Site	Latitude N	Longitude E	Site	Latitude N	Longitude E
W1	46° 10’ 08”	9° 46’ 11”	W6	46° 10’ 28”	9° 55’ 31”	W11	46° 09’ 11”	9° 59’ 59”
W2	46° 10’ 14”	9° 47’ 13”	W7	46° 09’ 40”	9° 59’ 29”	W12	46° 09’ 21”	9° 59’ 59”
W3	46° 10’ 24”	9° 47’ 23”	W8	46° 10’ 28”	9° 59’ 34”	W13	46° 10’ 01”	10° 00’ 29”
W4	46° 09’ 40”	9° 48’ 26”	W9	46° 09’ 26”	9° 59’ 36”	W14	46° 10’ 02”	10° 00’ 30”
W5	46° 09’ 58”	9° 50’ 26”	W10	46° 09’ 44”	9° 59’ 55”	W15	46° 10’ 09”	10° 00’ 30”

Table S3. 16SrV phytoplasma strains used in median-joining network analyses[a].

16SrV phytoplasma strain	*Map* genotype	Acc. No.	Host[b]	Country[c]
FDp strains	M3 (FD3)	AM384894	Vv, Ag	IT
(infecting grapevine and	M6 (FD3)	AM384895	Vv, Ag	IT
transmissible by *S. titanus)*	M12 (FD3)	AM384896	Vv, Oi	IT, RS, CH
	M27 (FD1)	AM384888	Vv, St	FR
	M34 (FD1)	AM384889	Vv	FR
	M38 (FD2)	LT221933	Vv, Ag, St, Oa, Oi, Al,	IT, FR, DE, HU, RS, HR
	M50 (FD1)	AM384887	Vv, Ag, Cv, St, Oa, Oi	IT, FR, DE, HU, CH
	M51 (FD3)	FN811141	Vv, Cv, Aa, St	IT, HU, RS, ME, HR
	M54 (FD2)	AM384886	Vv, Ca, St, Oi	IT, FR, CH, HR
	M89 (FD2)	OL830376	Vv	RS
	M112 (FD1)	LT222007	Vv	FR
	M119 (FD3)	FN811142	Cv	IT
	M121 (FD2)	LT222016	Vv, Oi, Al	FR
	M122 (FD2)	MG201984	Vv, St	FR, HU, CH
	M144 (FD3)	KJ911220	Vv, Cv	RS
	M145 (FD3)	KJ911221	Cv	ME
	M148 (FD2)	KJ605450	Vv, Ag	RS, ME
	M150 (FD3)	OL830381	Vv	RS
	M151 (FD3)	OL830379	Vv	RS
	M152 (FD3)	OL830377	Vv	RS
	M153 (FD3)	OL830368	Vv	RS
	M154 (FD3)	OL830370	Vv	RS
	M155 (FD2)	OL830366	Vv	RS
FDp-related strains	M36	AM384885	Vv, Ag	IT, FR
(PGY-associated phytoplasma,	M39	LT221934	Vv, Oa, Al	FR, DE
infecting grapevine but	M40	LT221935	Vv, Oa,	FR, DE, ME
non-transmissible by *S. titanus*)	M43	AM384890	Vv, Ag, Oa, Al	FR, DE, HU, ME
	M45	LT221940	Vv, Ag, Oa, Oi, Al,	FR, DE
	M46	AM384891	Vv, Ag, Oa	FR, DE
	M47	LT221942	Vv, Ag, Oa	FR, DE, HU, CH
	M48	AM384893	Vv, Ag, Oa, Al	FR, DE
	M52	LT221947	Vv, Ag, Oa, Al	FR, DE, HU, RS, MEO
	M53	AM384892	Vv, Ag, Oa, Al	IT, FR, DE, HU, RS, ME, HR
AldY-associated phytoplasma strains	M1	LT221896	Ag	FR
(never reported in grapevine;	M2	AM384897	Ag	FR
found in alder and insects)	M4	LT221899	Ag	DE
	M5	LT221900	Ag	FR
	M7	LT221902	Ag	DE
	M8	LT221903	Ag	DE
	M9	LT221904	Ag	DE
	M10	LT221905	Ag	FR
	M11	LT221906	Ag	DE
	M13	LT221908	Ag, Al	FR, DE
	M14	LT221909	Ag, Oa, Al	DE
	M15	LT221910	Ag	DE
	M16	LT221911	Oa	DE
	M17	LT221912	Ag	FR
	M18	LT221913	Ag	FR
	M19	LT221914	Ag	FR
	M20	LT221915	Ag	FR
	M21	LT221916	Oa	FR
	M22	LT221917	Ag	FR
	M23	LT221918	Ag	FR

AldY-associated phytoplasma strains	M24	LT221919	Ag	FR
(never reported in grapevine;	M25	LT221920	Ag	FR
found in alder and insects)	M26	LT221921	Ag	FR
	M28	LT221923	Ag	FR
	M29	LT221924	Ag	FR
	M30	LT221925	Ag	FR
	M31	LT221926	Ag	FR
	M32	LT221927	Ag	FR
	M33	LT221928	Ag	FR
	M35	AM384884	Ag	IT
	M36	AM384885	Ag	IT
	M37	FN561865	Ag	FR
	M41	LT221936	Ag, Oa	FR
	M42	LT221937	Ag	RS
	M44	LT221939	Ag, Oa	FR, DE, HU, CH
	M49	LT221944	Ag, Al	FR, DE, RS
	M55	LT221950	Ag	IT
	M56	LT221951	Ag	IT
	M57	LT221952	Ag	IT
	M58	LT221953	Ag	HU
	M59	LT221954	Ag	IT
	M61	LT221956	Ag	IT
	M62	LT221957	Ag	IT
	M63	LT221958	Oa	HU
	M64	LT221959	Ag	IT
	M65	LT221960	Ag	IT
	M66	LT221961	Ag	IT
	M67	LT221962	Ag	IT
	M68	LT221963	Ag	IT
	M69	LT221964	Ag	IT
	M70	LT221965	Ag	IT
	M71	LT221966	Ag	IT
	M72	LT221967	Ag	IT
	M73	LT221968	Ag	IT
	M74	LT221969	Ag	IT
	M75	LT221970	Ag	IT
	M76	LT221971	Ag	IT
	M77	LT221972	Ag	IT
	M78	LT221973	Ag, Oa, Al	FR, DE, RS
	M79	LT221974	Ag	DE
	M80	LT221975	Ag	DE
	M81	LT221976	Ag	DE
	M82	LT221977	Ag	DE
	M83	LT221978	Ag	DE
	M84	LT221979	Ag	DE
	M85	LT221980	Ag	DE
	M86	LT221981	Ag	DE
	M87	LT221982	Ag	DE
	M88	LT221983	Ag	RS
	M89	LT221984	Ag	RS
	M90	LT221985	Ag	RS
	M91	LT221986	Ag	RS
	M92	LT221987	Ag	RS
	M93	LT221988	Ag	RS
	M94	LT221989	Ag	RS
	M95	LT221990	Ag	RS

AldY-associated phytoplasma strains (never reported in grapevine; found in alder and insects)	M96	LT221991	Ag	RS
	M97	LT221992	Ag	RS
	M98	LT221993	Ag	RS
	M99	LT221994	Ag	RS
	M100	LT221995	Ag	IT
	M101	LT221996	Oa	DE, RS
	M102	LT221997	Ag	RS
	M103	LT221998	Ag	FR
	M104	LT221999	Ag	FR
	M105	LT222000	Ag	DE
	M106	LT222001	Ag	FR
	M107	LT222002	Oa	RS
	M108	LT222003	Oa	FR
	M109	LT222004	Oa	FR
	M110	LT222005	Oa	FR
	M111	LT222006	Ag	DE
	M113	LT222008	Ag	IT
	M114	LT222009	Oa	FR
	M115	LT222010	Oa	FR
	M116	LT222011	Ag, Oa	FR, DE
	M117	LT222012	Ag, Oa	FR, DE
	M118	LT222013	Ag	IT
	M120	LT222015	Oa	DE
	M123	LR585197	Oa	FR
	M124	LR585198	Oa	DE
	M125	LR585199	Oi	FR
	M126	LR585200	Oi	FR
	M127	LR585201	Oi	FR
	M129	LR585202	Oi	FR
	M130	LR585203	Oi	FR
	M131	LR585204	Oa	DE
	M132	LR585205	Al	FR
	M133	LR585206	Oa	FR
	M135	LR585207	Oa	DE
	M142	KC188998	Ag	ME
	M143	KC188999	Ag	ME
	M146	KJ605448	Ag	MK
	M147	KJ605449	Ag	MK
	M149	KJ605451	Ag	MK
	M156	OM654202	Ag	ME
	M157	OM654205	Ag	ME

[a] Utilized data were retrieved from references [7−11,19,20] listed in the manuscript.
[b] Host acronyms: plants (Aa, *Ailanthus altissima*; Ag, *Alnus glutinosa*; Ca, *Corylus avellana*; Cv, *Clematis vitalba*; Vv, *Vitis vinifera*); insects (Al, *Allygus spp.*; Oa, *Oncopsis alni*; Oi, *Orientus idhidae*; St, *Scaphoideus titanus*).
[c] Country acronyms: CH (Switzerland), DE (Germany), FR (France), HR (Croatia), HU (Hungary), IT (Italy), ME (Montenegro), MK (North Macedonia), RS (Serbia).
